# Urban and socioeconomic disparities in PM_2.5_ exposure across 340 Latin American cities

**DOI:** 10.1088/1748-9326/ae20a4

**Published:** 2025-12-02

**Authors:** Edson J Ascencio, Antony Barja, Jose Montes-Alvis, Josiah L Kephart, Nelson Gouveia, Daniel A Rodriguez, Tarik Benmarhnia, Ana V Diez Roux, Usama Bilal, J Jaime Miranda, Gabriel Carrasco-Escobar

**Affiliations:** 1 Health Innovation Laboratory, Institute of Tropical Medicine ‘Alexander von Humboldt’, Universidad Peruana Cayetano Heredia, Lima, Peru; 2 Urban Health Collaborative, Drexel Dornsife School of Public Health, Philadelphia, PA, United States of America; 3 Department of Environmental and Occupational Health, Drexel Dornsife School of Public Health, Philadelphia, PA, United States of America; 4 Department of Preventive Medicine, University of Sao Paulo Medical School, Sao Paulo, Brazil; 5 Department of City and Regional Planning and Institute of Transportation Studies, University of California, Berkeley, CA, United States of America; 6 Scripps Institution of Oceanography, University of California San Diego, La Jolla, CA, United States of America; 7 Department of Epidemiology and Biostatistics, Drexel Dornsife School of Public Health, Philadelphia, PA, United States of America; 8 CRONICAS Centre of Excellence in Chronic Diseases, Universidad Peruana Cayetano Heredia, Lima, Peru; 9 School of Medicine ‘Alberto Hurtado’, Universidad Peruana Cayetano Heredia, Lima, Peru; 10 Sydney School of Public Health, Faculty of Medicine and Health, University of Sydney, Sydney, NSW, Australia

**Keywords:** PM2.5, air pollution, Latin America, social disparities

## Abstract

*Background.* Fine particulate matter (PM_2.5_) is a leading global health risk. Latin American cities exhibit some of the world’s highest urban PM_2.5_ levels, yet studies of neighborhood-level PM_2.5_ exposure and associated disparities in the region are limited. *Methods.* We conducted a cross-sectional ecological analysis of 53 041 neighborhoods across 340 cities in eight Latin American countries, leveraging the Salud Urbana en America Latina study dataset. Annual PM_2.5_ concentrations were derived from satellite data and linked to socioeconomic and urban characteristics. A multilevel model analyzed associations between neighborhood PM_2.5_ levels and neighborhood- and city-level characteristics. *Results.* The median annual neighborhood PM_2.5_ concentration was 18.49 *µ*g m^−3^. Of the 256 million residents, all lived in neighborhoods with ambient PM_2.5_ concentrations that exceeded the 2021 World Health Organization guidelines (5 *µ*g m^−3^). Variability was greatest between cities (54.3% of total variance), but substantial within-city variation (26% of variance) was observed. Higher neighborhood PM_2.5_ levels were associated with higher neighborhood educational attainment (mean difference [MD] comparing top to bottom tertile = 0.17 *µ*g m^−3^), higher neighborhood intersection density (MD comparing top to bottom tertile = 0.17 *µ*g m^−3^), and older cities (MD comparing top to bottom tertile = 1.45 *µ*g m^−3^). Lower neighborhood PM_2.5_ levels were related to higher neighborhood population density (MD comparing top to bottom tertile = − 0.55 *µ*g m^−3^), more greenness (MD comparing top to bottom tertile = − 0.76 *µ*g m^−3^), and larger distance from city centers (MD comparing top to bottom tertile = − 0.86 *µ*g m^−3^). *Conclusions.* Neighborhoods with higher PM_2.5_ concentrations tended to have higher educational attainment, more intersections, and be located in older cities, while lower concentrations were associated with denser populations, more green space, and greater distance from city centers. Our findings reveal important within-city heterogeneity in PM_2.5_ and the factors associated with it, suggesting strategies to mitigate air pollution within cities.

## 1. Introduction

Outdoor air pollution related to suspended particles or particulate matter (PM) poses a significant environmental threat to human health, leading to 4.14 million deaths and 118.20 million disability-adjusted life years worldwide in 2019 alone [1]. Extensive research has consistently identified a strong association between PM_2.5_, i.e. fine particles less than 2.5 *μ*m in diameter, and increased morbidity related to cardiovascular disease, respiratory problems, and premature mortality [2–4].

Latin America stands out as one of the most urbanized regions globally, with around 80% of the total urban population in 2018 [5]. According to the World Air Quality Report 2024, Mexico, Peru, and Chile are alarmingly ranked among the top 60 most polluted countries worldwide [6]. The World Health Organization (WHO) estimates that in 2019, ambient air pollution led to approximately 175 846 deaths in Latin America [7]. Despite these staggering statistics, literature on PM_2.5_ exposure in Latin American cities is notably limited. The scarcity of ground monitors for measuring PM_2.5_ concentrations, even in major capitals, hampers the availability of high-quality and timely data [8, 9].

In characterizing social and urban disparities in pollutant exposure, it is crucial to explore small spatial scale variations such as those across urban neighborhoods within cities [10]. Focusing solely on larger areas may overlook social patterns of environmental exposures, particularly at small geographic scales, linked to residential segregation based on social class and race or ethnic background [11]. The Salud Urbana en America Latina study (SALURBAL) has meticulously compiled and harmonized health, social, and physical environment data for all cities with populations exceeding 100 000 in 2010 across 11 Latin American countries (Argentina, Brazil, Chile, Colombia, Costa Rica, El Salvador, Guatemala, Mexico, Nicaragua, Panama, and Peru) [12]. This unique dataset provides an opportunity to describe the relationship between PM_2.5_ exposure and urban and socioeconomic characteristics. Using SALURBAL data, we describe differences in PM_2.5_ concentrations across 53 041 neighborhoods in 340 Latin American cities. This information can provide a robust foundation to inform the design and recommendations of targeted air pollution control interventions in Latin America.

## 2. Methods

### 2.1. Study design

We conducted a cross-sectional multilevel analysis with ‘neighborhoods’ (the smallest unit with relevant data available in each country) nested within cities.

### 2.2. Study area and population

We focused on cities where PM_2.5_, socioeconomic, and urban data were accessible at the neighborhood level. Therefore, only cities in eight countries were included: Argentina, Brazil, Chile, Colombia, Costa Rica, Guatemala, Mexico, and Panama. Country-specific, small-area administrative units most comparable to US census tracts, hereafter denoted as ‘neighborhoods’, were used as proxies for urban neighborhoods within each city. Detailed information regarding the definitions of neighborhoods for each country can be found in table S1.

### 2.3. Data sources

#### 2.3.1. Urban and socioeconomic characteristics

We sourced socioeconomic data for neighborhoods from the latest national censuses conducted between 2002 and 2018 in each city, with details on census years provided in table S1. To ensure uniformity and comparability across cities, the socioeconomic and urban datasets for neighborhoods and cities underwent harmonization and processing procedures carried out by SALURBAL [12]. At the neighborhood level, we computed the population density, educational attainment, intersection density, greenness, and distance from the city center. Population (in thousands) density was computed per km^2^ of the neighborhood area. Educational attainment was defined as the percentage of the population aged 25 years or older who completed primary education or above. Intersection density was defined as the number of intersections per neighborhood area in km^2^. Intersections were extracted from street network OpenStreetMap data and included any intersections with >2 connected streets. Neighborhood area median greenness was measured by the normalized difference vegetation index (NDVI). NDVI was calculated using MODIS satellite-based observations from the MODIS vegetation product, MOD13Q1.006 for 2015 at a 250 m spatial resolution. We computed the maximum NDVI value for 2019 at 250 m resolution to present the ‘greenest’ condition of each grid cell within the year, then calculated the median across grid cells contained within each neighborhood. Distance from the city center was established as the Euclidean distance in km between the neighborhood centroid and city hall, based on neighborhood boundaries at the census time. At the city level, gross domestic product (GDP), population, and city age were computed. City-level GDP was originally calculated based on 2011 US dollars. Created by Gennaioli *et al* [13] in 2013 and converted into gridded estimates by Kummu *et al* [14] in 2015. GDP for each year from 1990 to 2015 was estimated by these authors, modeling data from government, surveys, and industry. Gridded estimates were matched to SALURBAL cities, and GDP was extracted directly from matching administrative units or using population-weighted averages in cases where city boundaries crossed multiple administrative areas. GDP estimates from 2015 (the most recent year available) were used. Population was established as the number of residents reported by the most recent available national census. City age was calculated as the number of years from the foundation year of each city to the year of each country’s census. SALURBAL used Encyclopedia Britannica, Wikipedia, and local or regional information websites as data sources for this variable. At least two matching data sources were required in order to establish the city foundation year.

#### 2.3.2. Ambient PM_25_

We computed the mean PM_2.5_ concentration for each neighborhood by aggregating data spanning the census year for each country, averaging it with the previous and following year of the census (a 3 year window). Annual mean PM_2.5_ concentrations were obtained from the Atmospheric Composition Analysis Group of Washington University of St. Louis (ATMOS) (https://sites.wustl.edu/acag/datasets/surface-pm2-5/) employing a spatial resolution of 0.01°× 0.01° (approximately 1.1 km by 1.1 km at the equator). Ground-level PM_2.5_ estimates were computed by combining aerosol optical depth retrievals (Dark Target, Deep Blue, and MAIAC) that use observations from multiple satellite-based NASA instruments (MODIS/Terra, MODIS/Aqua, MISR/Terra, SeaWiFS/SeaStar, VIIRS/SNPP, and VIIRS/NOAA20) with the GEOS-Chem chemical transport model (https://geos-chem.org), and subsequently calibrated to global ground-based observations using a geographically weighted regression [15, 16]. Finally, the data underwent further processing by the SALURBAL team [12], resulting in a measure of the 3 year window mean PM_2.5_ concentration for each neighborhood.

### 2.4. Data analysis

#### 2.4.1. Descriptive analysis

**Figure 1.**
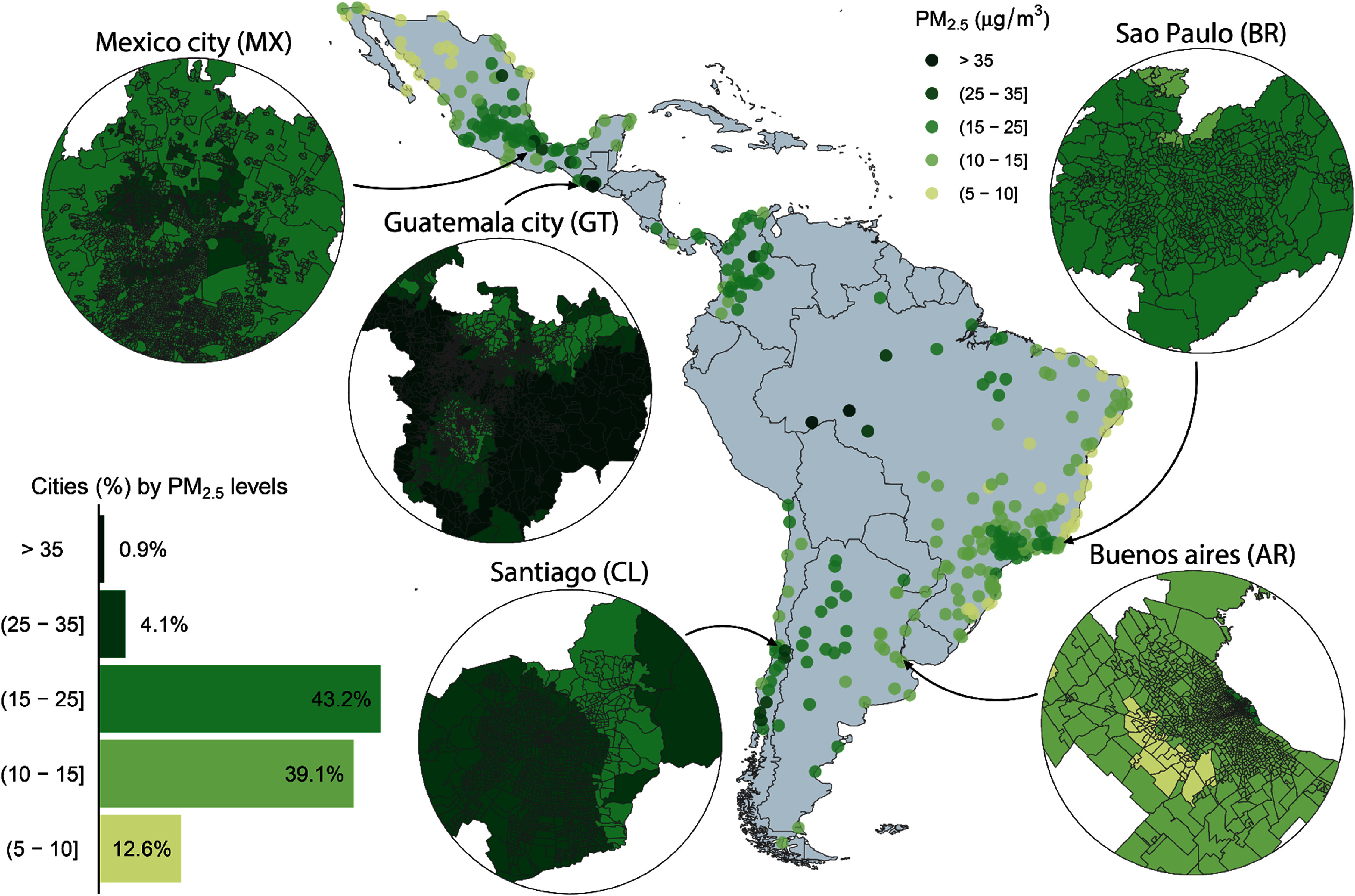
Location of study cities (*n* = 340) and three-year mean annual concentration of ambient PM_2.5_ for all study cities [DR2] and neighborhoods within select cities. The WHO guideline for annual PM_2.5_ is 5 *µ*g m^−3^. All neighborhoods that exceed this guideline are represented by green colors. *µ*g m^−3^ = micrograms per cubic meter. City-level PM_2.5_ was calculated using neighborhood PM_2.5_ (without population weights) with population weights per unit. Years used to estimate PM_2.5_ are 2002 ± 1 (Guatemala), 2010 ± 1 (Argentina, Brazil, Mexico, and Panama), 2011 ± 1 (Costa Rica), 2017 ± 1 (Chile), and 2018 ± 1 (Colombia).

Neighborhood characteristics were summarized using either the mean and standard deviation or the median and interquartile range (IQR). To facilitate visual interpretation, we categorized PM_2.5_ into the 2021 WHO PM_2.5_ guidelines and interim targets [17]. (figure 1).

#### 2.4.2. Multilevel model

We used a multilevel model (neighborhoods nested within cities within countries) to describe associations between city and neighborhood-level characteristics and neighborhood ambient PM_2.5_ concentrations. First, we examined Spearman’s correlations between all variables and found them moderate or low (ranging from −0.41 to 0.28 [figure S1]). We then fitted a fully adjusted model including all neighborhood and city-level variables with all predictors in categories based on tertiles of the observed distribution across the full sample. In supplementary analyses, we included predictors as *Z*-scores. All models included random intercepts for country and city. Finally, using the variance components from the multilevel model without predictors (null model), we estimated the percent of the variation in neighborhood PM_2.5_ between neighborhoods within cities, between cities within countries, and between countries.

## 3. Results

### 3.1. Characteristics of the study population

We examined 53 041 neighborhoods in 340 cities in eight Latin American countries (table 1). The geographical locations of observed cities are presented in figure 1. Overall, neighborhoods had a median population density of 5.66 thousand residents per km^2^ (IQR 2.00–11.21), a median NDVI (greenness) of 0.44 (0.30–0.62), and a median of 90% of the neighborhood population aged 25+ years had completed primary education (82.84–94.92). Neighborhoods in the pooled Central American group (Guatemala, Costa Rica, and Panama; median NDVI 0.59 [IQR 0.42–0.74]) and Brazil (0.57 [0.41–0.76]) were greenest, while neighborhoods in Chilean cities were the least green (0.32 [0.22–0.48]). Neighborhoods in Colombia had the highest population density (median 11.42 thousand residents per km^2^ [IQR 4.17–19.43]) while neighborhoods in Brazil were the least dense (3.90 [1.33–8.43]). At the city level, cities in Argentina had the greatest GDP per capita (median 19.5k USD [IQR 11.7–22.4]) while cities in Colombia had the lowest GDP (11.8k USD [8.6–13.8]). Neighborhood and city-level characteristics are described overall and by country in table 1.

**Table 1.** Characteristics of neighborhoods included in the analyses (53,041 neighborhoods within 340 Latin American cities in 8 countries). Data are *N* or median (IQR). NDVI = normalized difference vegetation index. GDP = gross domestic product. *Central American grouping includes urban neighborhoods in Costa Rica (*n* = 1 city), Guatemala (*n* = 3), and Panama (*n* = 3).

	Total	Argentina	Brazil	Central America*	Chile	Colombia	Mexico

Neighborhood level							
Number of neighborhoods	53 041	2 409	4 023	5 988	3 913	3 781	32 927
Population density (1000 people per km^2^)	5.66 (2.00, 11.21)	5.70 (1.82, 9.12)	3.90 (1.33, 8.43)	5.98 (2.02, 14.28)	8.03 (3.63, 13.26)	11.42 (4.17, 19.43)	5.22 (1.92, 10.17)
Percent persons >25 years with complete primary or more	89.99 (82.84, 94.92)	90.03 (84.00, 95.30)	67.37 (60.25, 75.10)	78.87 (58.35, 90.74)	91.55 (86.21, 95.77)	88.34 (80.55, 93.32)	91.68 (87.11, 95.90)
Intersection density per km^2^	129.90 (67.58, 199.00)	86.45 (51.34, 107.91)	88.28 (38.11, 128.79)	74.40 (18.19, 146.71)	148.44 (81.48, 230.02)	101.05 (20.20, 304.51)	151.58 (92.36, 211.61)
Greenness (NDVI)	0.44 (0.30, 0.62)	0.40 (0.30, 0.60)	0.57 (0.41, 0.76)	0.59 (0.42, 0.74)	0.32 (0.22, 0.48)	0.54 (0.40, 0.72)	0.41 (0.28, 0.57)
Distance from city center (km)	8.42 (4.23, 15.76)	14.59 (5.37, 29.43)	10.29 (4.45, 19.90)	9.36 (5.68, 14.83)	8.01 (3.60, 13.68)	5.03 (2.46, 10.25)	8.23 (4.31, 15.66)

City level							

Number of cities	340	33	152	7	21	35	92
City population (thousands)	309.81 (183.33, 642.03)	310.12 (213.26, 583.51)	256.73 (165.53, 572.68)	309.49 (239.29, 2,378.30)	247.55 (176.48, 385.04)	355.71 (176.72, 620.95)	399.58 (237.80, 896.58)
GDP per capita (US$ thousands)	14.78 (10.47, 20.63)	19.59 (11.75, 22.42)	19.26 (8.60, 20.99)	14.47 (9.65, 22.41)	17.69 (13.01, 26.56)	11.82 (8.62, 13.82)	13.96 (11.47, 17.62)
Population density	4.28 (2.84, 6.95)	3.36 (2.97, 4.21)	2.80 (2.03, 3.65)	6.08 (4.60, 9.06)	5.93 (5.56, 7.13)	10.78 (8.96, 14.63)	6.21 (5.11, 7.72)
Education	80.89 (65.60, 88.83)	88.08 (85.91, 89.59)	65.16 (61.14, 69.05)	85.83 (56.29, 88.89)	89.82 (88.04, 91.51)	83.56 (80.70, 85.94)	89.49 (87.55, 90.88)
Intersection density per km^2^	94.18 (63.91, 142.33)	80.32 (66.11, 95.67)	67.58 (51.57, 86.24)	57.52 (56.47, 85.98)	154.54 (146.07, 170.98)	152.53 (97.93, 200.89)	145.47 (117.82, 166.34)
Greenness (NDVI)	0.58 (0.50, 0.65)	0.50 (0.43, 0.56)	0.64 (0.59, 0.70)	0.63 (0.58, 0.70)	0.45 (0.28, 0.56)	0.61 (0.56, 0.65)	0.47 (0.37, 0.56)

### 3.2. PM_2.5_ exposure distribution across cities and countries

Our study area included approximately 256 million residents as of the most recent census, ranging from 7 million residents in Central America to 108.8 million residents in Brazil (table 2). Of these 256 million residents, 100% lived in neighborhoods with annual ambient PM_2.5_ concentrations above 2021 WHO guidelines [17] (5 *µ*g m^−3^), and 91.9% (nearly 235 million people) lived in neighborhoods with annual ambient PM_2.5_ concentrations above pre-2021 WHO guidelines (10 *µ*g m^−3^). The percentage of residents living with ambient PM_2.5_ concentrations above pre-2021 WHO guidelines varied from 86.0% of residents (93.6 million of 108.8 million) in Brazilian cities to 100% of residents in Argentinian (27.6 million), Central American (7 million), and Colombian (26.8 million) cities.

**Table 2.** Neighborhood ambient PM_2.5_ concentrations and population exposures among 53 041 study neighborhoods in 340 Latin American cities. *Mean value across three years centered at the year of the census used to derive neighborhood features (census years are 2002 [Guatemala], 2010 [Argentina, Brazil, Mexico, and Panama], 2011 [Costa Rica], 2017 [Chile], and 2018 [Colombia]). *Central American grouping includes urban neighborhoods in Costa Rica (*n*= 1 city), Guatemala (*n* = 3), and Panama (*n* = 3).

	Study population (millions)	Study population above PM_2.5_ 2021 guidelines (millions)	Percentage of study population above PM_2.5_ 2021 guidelines	Study population above PM_2.5_ pre-2021 guidelines (millions)	Percentage of study population above PM_2.5_ pre-2021 guidelines	Mean neighborhood PM_2.5_ (*µ*g m^−3^)	5th percentile neighborhood PM_2.5_ (*µ*g m^−3^)	Median neighborhood PM_2.5_ (*µ*g m^−3^)	95th percentile neighborhood PM_2.5_ (*µ*g m^−3^)
Argentina	27.65	27.65	100.00	27.65	100.00	14.81	10.80	13.48	22.76
Brazil	108.85	108.85	100.00	93.61	86.00	14.57	8.44	13.72	21.60
Central America*	7.01	7.01	100.00	7.01	100.00	27.93	14.30	22.94	50.77
Chile	12.29	12.29	100.00	12.29	99.99	23.12	13.00	25.55	30.12
Colombia	26.84	26.84	100.00	26.84	100.00	17.68	13.99	17.08	22.30
Mexico	73.96	73.96	100.00	68.52	92.65	17.73	7.79	18.77	25.77

Total	256.60	256.60	100.00	235.92	91.94	18.90	8.59	18.49	29.13

**Figure 2.**
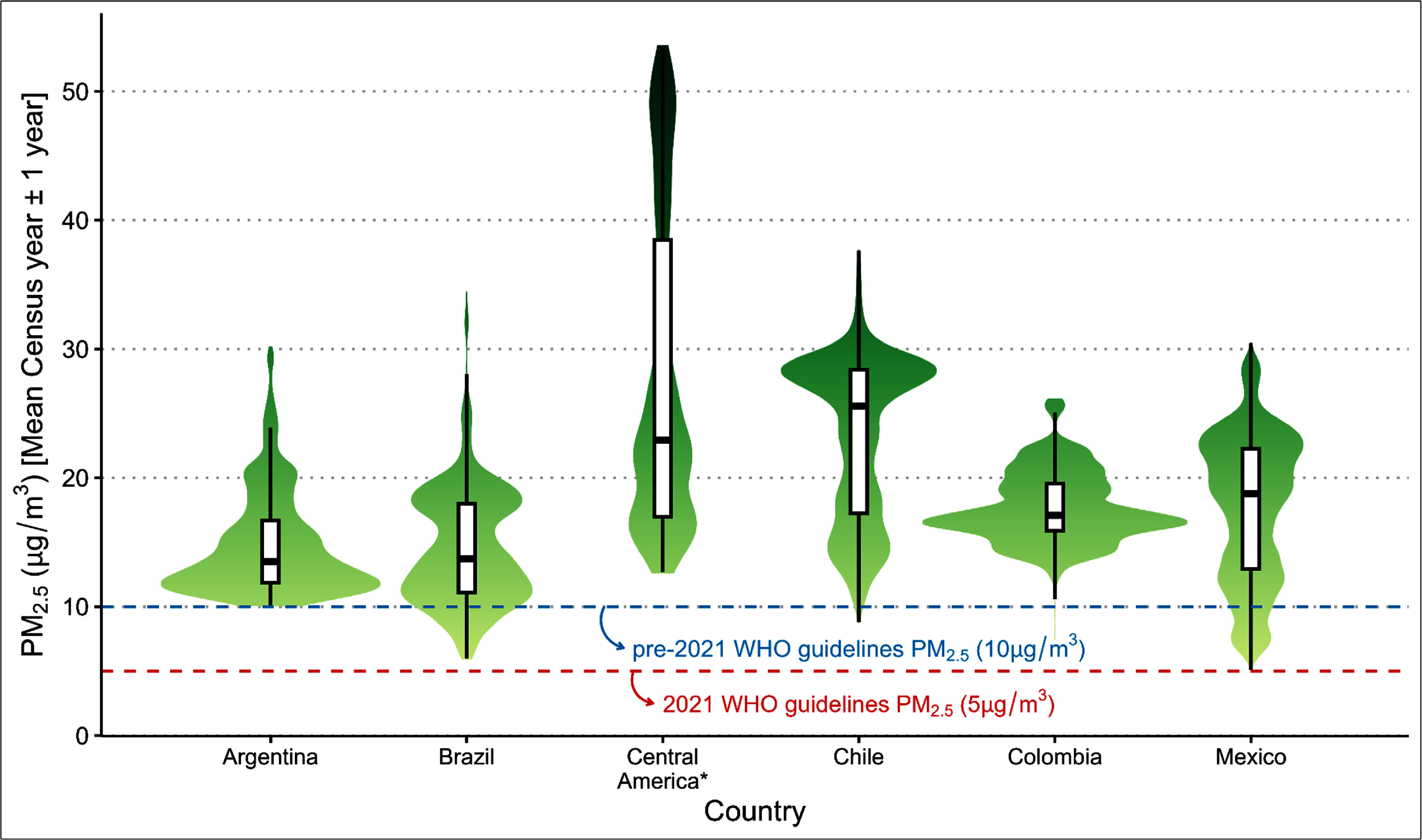
Annual ambient PM_2.5_ within 53 041 urban neighborhoods in Latin America. Violin plots show the distribution of neighborhoods by annual PM_2.5_ levels in each country (the thicker the plot horizontally the more neighborhoods at that PM_2.5_ level). The overlapped boxplots show the median and interquartile range. The red dashed line represents the 2021 WHO guidelines for annual PM_2.5_ (5 *µ*g m^−3^). The blue dashed line represents the pre-2021 guideline for annual PM_2.5_ (10 *µ*g m^−3^). *Central American grouping includes urban neighborhoods in Costa Rica (*n* = 1 city), Guatemala (*n* = 3), and Panama (*n* = 3).

In figure 1, we present the city-level mean PM_2.5_ for context, and five cities to illustrate the neighborhood-level concentrations. The median neighborhood annual PM_2.5_ *µ*g m^−3^ across all countries was 18.49 *µ*g m^−3^ (IQR 13.67–22.53), nearly four times the WHO annual guideline of 5 *µ*g m^−3^ (table 2). Median neighborhood PM_2.5_ varied between countries, ranging from 13.48 *µ*g m^−3^ in Brazil to 25.55 *µ*g m^−3^ in Chile. Cities with the highest PM_2.5_ concentrations per country were San Carlos de Bariloche (Argentina) with 24.22 *µ*g m^−3^, Porto Velho (Brazil) with 35.98 *µ*g m^−3^, Temuco (Chile) with 33.39 *µ*g m^−3^, Barrancabermeja (Colombia) with 25.99 *µ*g m^−3^, San Jose (Costa Rica) with 20.11 *µ*g m^−3^, Escuintla (Guatemala) with 42.19 *µ*g m^−3^, Tuxtla Gutierrez (Mexico) with 27.95 *µ*g m^−3^, and Panama City (Panama) with 15.99 *µ*g m^−3^. We observed substantial variation in PM_2.5_ concentrations across neighborhoods within countries (table 2 and figure 2). For example, in Central America, the 5th percentile neighborhood PM_2.5_ concentration was 14.3 *µ*g m^−3^ while the 95th percentile concentration was 50.7 *µ*g m^−3^.

The distribution of cross-classified tertiles of PM_2.5_ concentrations and tertiles of the population aged 25+ years who had completed primary education (figure S1). The distribution of these cross-classified categories had multiple patterns. For example, in Guatemala, the population that experienced the highest tertile of PM_2.5_ concentrations and the lowest tertile of education was located in the peripheral area of the city, while the population that experienced the lowest tertile of PM_2.5_ concentration and the highest tertiles of education was located in the main central area of the city. In contrast in Mexico City, neighborhoods with high PM_2.5_ and low education were in the south of the city while those with and low PM_2.5_ and high education were located in the north of the city.

### 3.3. Multilevel models

**Figure 3.**
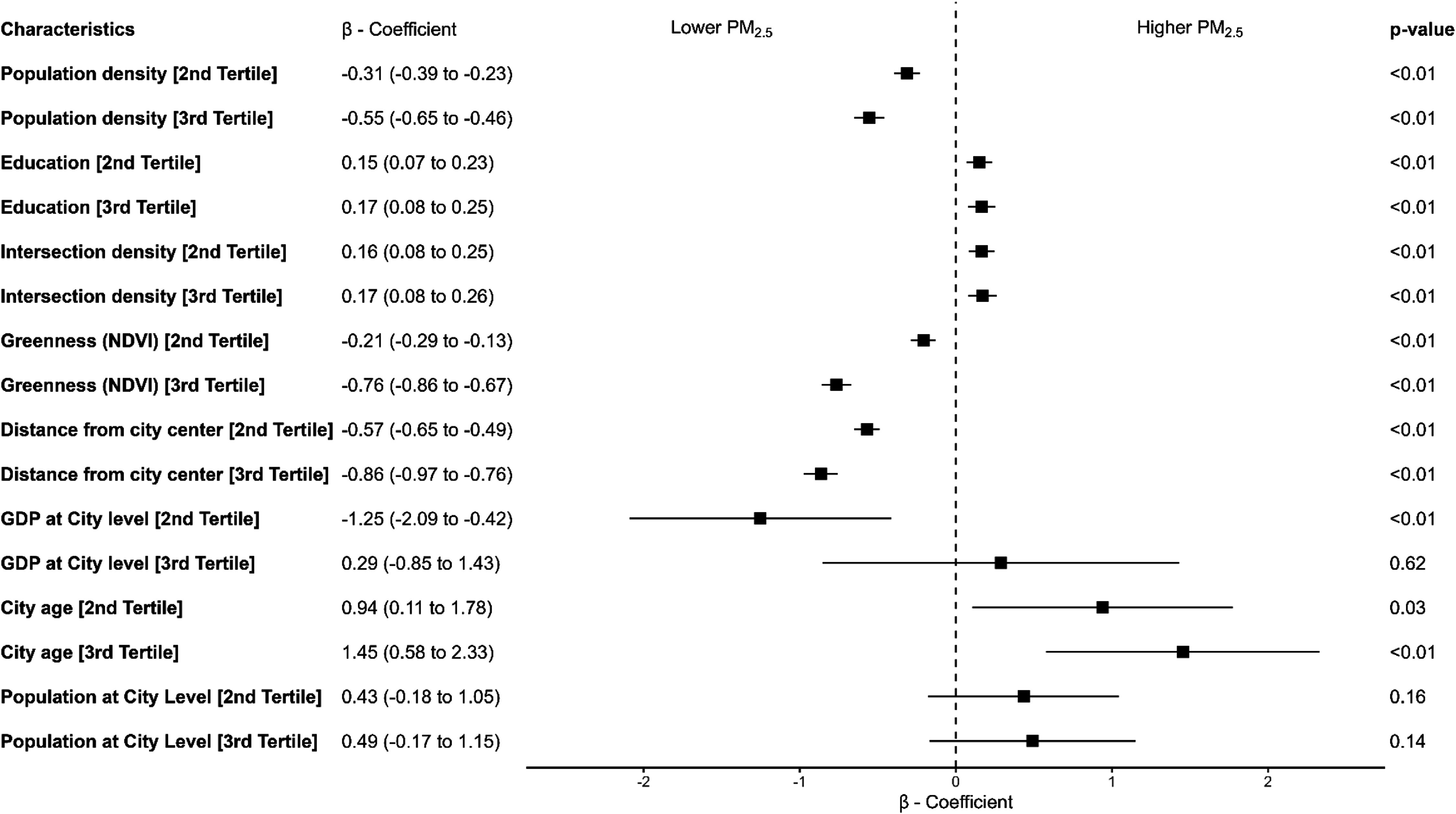
Associations of neighborhood-level and city-level features with neighborhood ambient PM_2.5_ *µ*g m^−3^ in 53 041 urban neighborhoods in Latin America. Multivariable model with a random intercept for city and country. We included all neighborhood and city-level variables with all predictors in categories based on tertiles of the observed distribution across the full sample. The *β*-coefficient represents the MD in PM_2.5_ comparing the specified tertile to the bottom tertile of the variable.

Figure 3 shows the associations between neighborhood and city-level characteristics and neighborhood PM_2.5_ ambient concentrations. Spearman correlation coefficients for all variables are presented in figure S2, showing correlation coefficients ranging from −0.41 to 0.28. The analysis of variance components (null model) showed that 20.1% of the total variance in neighborhood PM_2.5_ was between countries, 54.3% was between cities within countries, and 25.6% was between neighborhoods within cities.

In the fully-adjusted model including neighborhood- and city-level characteristics simultaneously in tertiles, higher educational attainment tertile (mean difference [MD] = 0.15 *µ*g m^−3^), and higher intersection density tertile (MD = 0.17 *µ*g m^−3^) were associated with higher neighborhood PM_2.5_ compared to the first tertile of each characteristic. Conversely, lower neighborhood PM_2.5_ was associated with higher population density tertile (MD = − 0.55 *µ*g m^−3^), greater distance from the city center tertile (MD = − 0.86 *µ*g m^−3^), and more neighborhood greenness tertile (MD = − 0.76 *µ*g m^−3^) compared to the first tertile of each characteristic. Additionally, a higher city-level age tertile (MD = 1.45 *µ*g m^−3^) was associated with higher neighborhood PM_2.5_ compared to the first tertile of city age (figure 3).

In the supplementary analysis, the *Z*-score covariate model (table S2) evidenced that higher educational attainment (0.12 higher PM_2.5_ *µ*g m^−3^ per unit higher education *Z*-score [95% CI 0.08–0.16]) was associated with higher neighborhood PM_2.5_. Conversely, lower neighborhood PM_2.5_ was associated with higher neighborhood population density (−0.32 *µ*g m^−3^ per unit higher population density *Z*-score [95% CI −0.35 to −0.29]), more neighborhood greenness (−0.38 *µ*g m^−3^ per unit NDVI *Z*-score [−0.42 to −0.34]) and greater distance from the city center (−0.18 *µ*g m^−3^ per unit distance *Z*-score [−0.22 to −0.14]).

## 4. Discussion

This study explored the associations of PM_2.5_ concentrations with urban and socioeconomic conditions in 340 Latin American cities, collectively home to almost 256 million residents. First, all residents are exposed to ambient PM_2.5_ concentrations (3 year window) that exceed current WHO guidelines, and nearly nine out of ten residents (∼235 million people) are exposed to concentrations that exceed pre-2021 WHO guidelines. Second, we found that of the total variability in neighborhood PM_2.5_ levels, the largest proportion was between cities within countries, although some within-city variation was also observed. Third, we found that neighborhoods with lower population density, less vegetation, closer to the city center, higher neighborhood intersection density, and higher educational attainment had higher concentrations of PM_2.5_. The fine granularity of our observations at the neighborhood level afforded precise estimates, providing a robust foundation to inform the design of targeted air pollution control interventions. This, in turn, aids in identifying the most PM_2.5_-exposed populations, underscoring the significance of context-specific environmental health research.

Given the 50% reduction in annual PM_2.5_ guidelines in the 2021 WHO air quality guidelines (from 10 to 5 *µ*g m^−3^), urban populations worldwide find themselves with long-term PM_2.5_ concentrations above the updated guidelines established to protect public health. Our highly spatially granular analysis of urban Latin America finds that Latin American cities are no exception, with 100% (256 million) residents in our study area living in neighborhoods with PM_2.5_ concentrations above 2021 WHO guidelines.

Understanding the drivers of between-city and within-city differences in ambient PM_2.5_ exposure is critical to designing policies that promote health and health equity in this highly urbanized region [18]. Our study is unique in its breadth (all cities of 100 000 residents or more in eight countries) and in our examination of how neighborhood and city-level factors independently relate to urban PM_2.5_ exposure. In models adjusted for city and neighborhood-level factors, we found that greenness at the neighborhood level and more distance from the city center was associated with lower neighborhood PM_2.5_. This is unsurprising given the continued dominance of fossil fuels for motor vehicles and transit, and the role of fossil fuel motorized transit in generating PM_2.5_ [19, 20]. Overall, our findings suggest that increasing neighborhood greenness warrants further attention as a potential actionable environmental intervention to reduce population exposure to PM_2.5_ in urban areas.

Studies of diverse cities have found varying and even opposite associations between population density and ambient PM_2.5_, likely reflecting heterogeneous relationships over place and time between population density and transportation features such as mode (active transport vs. automobile) and congestion. In some settings, higher density is linked to lower PM_2.5_, consistent with reduced vehicle travel and greater public transit use. For example, Bereitsnaft *et al* [21] found that among 86 metropolitan areas in the US, low-density urban sprawl was associated with higher concentrations of air pollution. In contrast, other analyses find higher PM_2.5_ in denser cities. In Shanghai, Han *et al* [22]. Reported that more densely populated sub-districts had higher PM_2.5_. In some contexts, the relationship between urban density and PM_2.5_ was non-linear, often inverted-U shaped. Kim *et al* [23] found that moderate density was associated with lower PM₂.₅, while both low-density sprawl and very high density showed higher PM_2.5_ concentrations. The association of population density with PM_2.5_ may differ depending on the spatial scale examined. Notably in earlier SALURBAL analyses Gouveia *et al* [18] found that Latin American cities with higher population densities had lower PM_2.5_ levels. In contrast, the analyses reported here using smaller areas suggested the opposite. It is plausible that, at the city level, higher population density is associated with lower PM_2.5_ concentrations due to the relationship between density, transportation infrastructure, and collective mobility patterns. However, at smaller spatial scales, the relationship may be reversed, as high-density neighborhoods are often situated in areas with greater traffic congestion, leading to elevated local emissions.

In contrast to population density, international literature more uniformly links higher intersection density to higher PM_2.5_. Intersection density is widely used as a proxy for street connectivity and the intensity of stop-and-go driving. In Shanghai, the proportion of roads that were intersections had a strongly positive effect on PM_2.5_[22]. A SALURBAL analysis of Latin American cities found that moderate to high street connectivity was associated with higher PM_2.5_, as these patterns imply frequent stopping and slow traffic[24]. Likewise, Gouveia *et al* reported that within Latin American cities, sub-areas with more intersections had higher PM_2.5_[18]. Each additional intersection could add more idling time and acceleration cycles for vehicles and generate non-tailpipe particulate emissions from brakes and tires [22].

Notably, we also observed higher PM_2.5_ levels in higher education neighborhoods, a pattern that is the opposite of what has been reported in high-income countries [25, 26]. This is similar to what was also reported for NO_2_ in SALURBAL [10]. This may reflect patterns of residential segregation in Latin American cities, by which higher socioeconomic areas are often concentrated in city centers with more traffic and greater intersection density. However, as residential segregation evolves, these patterns may be changing over time. Of note the positive association that we observed between neighborhood education and PM_2.5_ was weak and may mask substantial heterogeneity in this association across cities and over time.

We evidenced that higher neighborhood PM_2.5_ was associated with high education and intersection density and proximity to city centers, whereas denser, greener, and peripheral neighborhoods had lower PM_2.5_. A similar pattern was described in Taiwan, where core metropolitan townships—which are also higher-SES—had the highest PM_2.5_, in contrast to remote rural districts [27]. However, some Asian cities differed: A positive association was observed between PM_2.5_ and SES in Delhi, while a negative association was observed in Chennai [28]. In East Africa, the opposite was observed: city centers had lower PM_2.5_ than suburban slums, likely because of biomass burning and loss of vegetation in peri-urban zones drove pollution spikes [29]. In most settings, more vegetation generally coincided with lower PM_2.5,_ while the effect of population and SES varied across cities.

Our findings of greater variability in PM_2.5_ between cities than within cities underscore the importance of centralized city- and regional-level policies to reduce PM_2.5_ concentrations across large urban areas. While there are likely hyperlocal hotspots in PM_2.5_ concentrations, such as power plants or factories, which may bring environmental justice concerns for nearby communities, our analysis was unable to detect these local hotspots because our spatial resolution for PM_2.5_ was limited to 1.1 km level or greater.

It is important to acknowledge the limitations inherent in this study. Our analysis relied on static measures of ambient PM_2.5_, assuming that individuals are exposed to the PM_2.5_ levels in the neighborhood where they reside. However, the dynamic nature of exposure, influenced by human mobility, e.g., commuting to work during the day, returning home at night, or traveling to different regions for extended periods, implies that levels of population exposure may be somewhat different from those reported here [30]. Even so, individuals do spend a significant amount of time in their neighborhoods, making these analyses informative. Furthermore, Mexico comprises 62.07% of the neighborhoods, yet only 27.05% of the cities, indicating a disproportionate concentration of neighborhoods within a relatively limited number of urban areas. This country exhibits a mean population density of 5.22 thousand inhabitants per km^2^, the second lowest among the countries analyzed, surpassed only by Brazil. This pattern underscores the relatively sparse distribution of population within Mexico’s urban regions. To partially address this limitation, our analyses were adjusted by both population density and the distance of neighborhoods to the city center, thereby accounting for variations in urban structure and improving the comparability of results across countries. In addition, future studies of the SALURBAL consortia will address cross-level interactions and random slopes of the socioeconomic variables. Finally, it is important to highlight that a major strength of this study is the inclusion of a large number of neighborhoods, encompassing all cities with populations of 100 000 inhabitants or more, which enhances the robustness and generalizability of our findings.

## 5. Conclusions

Our study revealed that among 256 million residents in 340 Latin American cities, all live in neighborhoods with ambient PM_2.5_ concentrations that exceed 2021 WHO guidelines, and nearly nine out of ten people live in neighborhoods with ambient PM_2.5_ concentrations that exceed pre-2021 WHO guidelines. Neighborhoods that have lower population density, higher intersection density, are closer to the urban core, have higher educational attainment, and are less green have higher concentrations of PM_2.5_. Our findings are important evidence at the neighborhood level to better inform public policies and interventions to mitigate air pollution within cities.

## Data Availability

Socioeconomic data were obtained directly from statistical agencies in each country. A link to these agency websites can be accessed at https://drexel.edu/lac/data-evidence/data-acknowledgements. The SALURBAL project welcomes queries from anyone interested in learning more about its dataset and potential access to data. To learn more about SALURBAL’s dataset, visit https://drexel.edu/lac/ or contact the project at salurbal@drexel.edu. Please contact the corresponding author about access to neighborhood-level urban features. Supplementary material available at https://doi.org/10.1088/1748-9326/ae20a4/data1.

## Code availability

Code is available at the following link: https://github.com/healthinnovation/airpollution_latam.

## References

[1] Sang S, Chu C, Zhang T, Chen H, Yang X (2022). The global burden of disease attributable to ambient fine particulate matter in 204 countries and territories, 1990–2019: a systematic analysis of the global burden of disease study 2019. Ecotoxicol. Environ. Saf..

[2] Newman J D (2020). Cardiopulmonary impact of particulate air pollution in high-risk populations: JACC state-of-the-art review. J. Am. Coll. Cardiol..

[3] Waidyatillake N T, Campbell P T, Vicendese D, Dharmage S C, Curto A, Stevenson M (2021). Particulate matter and premature mortality: a Bayesian meta-analysis. Int. J. Environ. Res. Public Health.

[4] Chen J, Hoek G (2020). Long-term exposure to PM and all-cause and cause-specific mortality: a systematic review and meta-analysis. Environ. Int..

[5] United Nations Department of Economic and Social Affairs (2019). World urbanization prospects 2018: highlights.

[6] IQAir (2022). 2022 world air quality report. Reg City PM2.

[7] World Health Organization (2017). Burden of Disease from Ambient Air Pollution for 2016 Description of Method V5 May 2018.

[8] Fajersztajn L, Saldiva P, Pereira L A A, Leite V F, Buehler A M (2017). Short-term effects of fine particulate matter pollution on daily health events in Latin America: a systematic review and meta-analysis. Int. J. Public Health.

[9] Grisales-Romero H, Piñeros-Jiménez J G, Nieto E, Porras-Cataño S, Montealegre N, González D, Ospina D (2021). Local attributable burden disease to PM 2.5 ambient air pollution in Medellín, Colombia, 2010–2016. F1000Research.

[10] Kephart J L, Gouveia N, Rodríguez D A, Indvik K, Alfaro T, Texcalac-Sangrador J L, Miranda J J, Bilal U, Diez Roux A V (2023). Ambient nitrogen dioxide in 47 187 neighbourhoods across 326 cities in eight Latin American countries: population exposures and associations with urban features. Lancet Planet. Health.

[11] Clark L P, Harris M H, Apte J S, Marshall J D (2022). National and intraurban air pollution exposure disparity estimates in the united states: impact of data-aggregation spatial scale. Environ. Sci. Technol. Lett..

[12] Quistberg D A (2019). Building a data platform for cross-country urban health studies: the SALURBAL study. J. Urban Health..

[13] Gennaioli N, La Porta R, Lopez-de-silanes F, Shleifer A (2013). Human capital and regional development *. Q. J. Econ..

[14] Kummu M, Taka M, Guillaume J H A (2018). Gridded global datasets for gross domestic product and human development index over 1990–2015. Sci. Data.

[15] van Donkelaar A (2021). Monthly global estimates of fine particulate matter and their uncertainty. Environ. Sci. Technol..

[16] Hammer M S (2023). Assessment of the impact of discontinuity in satellite instruments and retrievals on global PM_2.5_ estimates. Rem. Sens. Environ..

[17] World Health Organization (2021). WHO Global Air Quality Guidelines: Particulate Matter (PM2.5 And PM10), Ozone, Nitrogen Dioxide, Sulfur Dioxide and Carbon Monoxide.

[18] Gouveia N (2021). Ambient fine particulate matter in Latin American cities: levels, population exposure, and associated urban factors. Sci. Total Environ..

[19] Hao Y, Gao C, Deng S, Yuan M, Song W, Lu Z, Qiu Z (2019). Chemical characterisation of PM_2.5_ emitted from motor vehicles powered by diesel, gasoline, natural gas and methanol fuel. Sci. Total Environ..

[20] Lim S (2020). Fossil-driven secondary inorganic PM_2.5_ enhancement in the North China plain: evidence from carbon and nitrogen isotopes. Environ. Pollut..

[21] Bereitschaft B, Debbage K (2013). Urban form air pollution, and CO_2_ emissions in large U.S. metropolitan areas. Prof. Geogr..

[22] Han S, Sun B (2019). Impact of population density on PM_2.5_ concentrations: a case study in Shanghai, China. Sustainability.

[23] Kim M-J, Chang Y-S, Kim S-M (2021). Impact of income, density, and population size on PM_2.5_ pollutions: a scaling analysis of 254 large cities in six developed countries. Int. J. Environ. Res. Public Health.

[24] Sarmiento O L (2021). Built environment profiles for Latin American urban settings: the SALURBAL study. PLoS One.

[25] Castillo M D, Kinney P L, Southerland V, Arno C A, Crawford K, Van donkelaar A, Hammer M, Martin R V, Anenberg S C (2021). Estimating intra-urban inequities in PM_2.5_-attributable health impacts: a case study for Washington, DC. GeoHealth.

[26] Mustansar T, Timmermans E J, Silva A I, Bijnens E M, Lefebvre W, Saenen N D, Vanpoucke C, Nawrot T S, Vaartjes I (2025). Socioeconomic inequalities and ambient air pollution exposure in school-aged children living in an affluent society: an analysis on individual and aggregated data in Belgium. Health Place.

[27] Lin P-Y, Lo Y-Y, Lin W-Y, Wu C-D, Liang W-M, Kuo H-W (2025). Urban–rural disparity for socioeconomic inequality regarding PM_2.5_ exposure. Aerosol Air Qual. Res..

[28] Menon J S, Mandal S, Ali M K, Deepa M, Mohan V, Schwartz J D, Prabhakaran D, Prabhakaran P (2025). The role of neighborhood and household level socio-economic status in determining air pollution exposure in two Indian cities. Environ. Res. Lett..

[29] Chua S D X (2025). East African city centers show lower PM_2.5_ levels than their suburbs. Environ. Sci. Technol. Lett..

[30] Sarmiento O L (2020). Urban transformations and health: methods for TrUST—a natural experiment evaluating the impacts of a mass transit cable car in Bogotá, Colombia. Front. Public Health.

